# Bariatric Surgery in Patients with Existing Ostomy: A Preliminary Feasibility Study

**DOI:** 10.1089/bari.2021.0131

**Published:** 2022-06-08

**Authors:** Ray Portela, Ahmet Vahibe, Joseph N. Badaoui, Omer U.I. Hassan, Travis J. Mckenzie, Todd A. Kellogg, Omar M. Ghanem

**Affiliations:** Department of Surgery, Mayo Clinic, Rochester, Minnesota, USA.

**Keywords:** obesity, bariatrics, gastrointestinal, minimally invasive surgery

## Abstract

**Background:**

Bariatric surgery is the most effective treatment for weight loss and obesity-related comorbidity resolution. However, bariatric surgery is not readily offered in specific populations due to the lack of data assessing its feasibility. This study intends to evaluate bariatric surgery in patients with an existing ostomy.

**Methods:**

We conducted a retrospective case series to assess the safety of Roux-en-Y gastric bypass (RYGB) or sleeve gastrectomy (SG) in patients with an end ileostomy or colostomy. Patient demographics, including obesity-related comorbidities, overall health status (American Society of Anesthesiologists score), and short-term complications (up to 30 days postoperatively), were analyzed.

**Results:**

Six patients were included. The mean age was 58 years, and the mean preoperative body mass index was 41.6. Three patients had a colostomy, and three had an ileostomy. The mean time of ostomy before surgery was 11 years. Two ostomies were due to trauma, two due to inflammatory bowel disease, one due to cancer, and one due to scleroderma. Mean postoperative follow-up was 23 months. No patient had increased ostomy output or infusion center visit. One patient had an ED visit, one had a short-term complication, and one had SG conversion to RYGB.

**Conclusions:**

Bariatric surgery is technically feasible in selected patients with ileostomy/colostomy with a reasonable short-term safety profile.

**KEY POINTS**

1. Feasible and safe to perform bariatric procedures in selected patients with an ileostomy or colostomy.

2. Patients with preexisting ostomy neither have a higher incidence of dehydration nor have an increased ostomy output after bariatric surgery.

## Introduction

The obesity epidemic affects more than 1 billion people worldwide and is one of the major contributors to health care costs.^1^ Bariatric surgery is proven to be the most effective and durable treatment for obesity.^2,3^ Roux-en-Y gastric bypass (RYGB) and sleeve gastrectomy (SG) are the most common procedures performed in the United States.^4–6^ Both RYGB and SG are offered to a wide array of patients since the risk-benefit profile associated with these procedures is usually favorable. However, particular situations may arise where the safety and feasibility of bariatric surgery have not been studied enough. This lack of data in the scientific literature puts the bariatric surgeon in a challenging position if they were ever faced with a particular situation; an example, patients with an existing ostomy who also suffer from obesity.

Are they adequate surgical candidates? Patients with stoma history have undergone previous abdominal surgeries and thus are believed to have higher complication rates.^6,7^ In addition, a certain bowel length is bypassed in RYGB, and there is increased gastric emptying in sleeve patients^8,9^; two variables that can potentially lead to higher ostomy output and eventually dehydration. However, should this be enough to deprive these patients of a bariatric procedure that can positively impact their overall health and quality of life? Beyond one case report portraying safety, there exist no data on patients undergoing bariatric surgery in the setting of pre-existing ostomy.^10^ In this series, we intend to assess the technical feasibility and safety profile of bariatric surgery in patients with a previous colostomy or ileostomy.

## Methods

### Study design and population

After Institutional Review Board approval, patients from January 1, 2015, to May 31, 2021, with a pre-existing ostomy before bariatric surgery were included in this retrospective case series spanning across the Mayo Clinic Enterprise.

The inclusion criteria were age ≥18 years and presence of an ileostomy or colostomy before bariatric surgery. Colostomy was identified using the International Classification of Diseases (ICD) 10 code Z93.3 and ileostomy using the ICD 10 code Z93.2. Not to miss any patients, all stomach and bypass procedures were included and identified using the Current Procedural Terminology (CPT) codes 43621, 43632, 43633, 43644, 43645, 43659, 43774, 43775, 43840, 43846, 43848, 43860, 43887, and 43999. Patients were observed for short- and long-term complications.

### Surgical technique

The SG was tailored using a bougie (40-F) starting 4–6 cm proximal to the pylorus and toward the stomach body until about 0.5–1 cm away from the angle of His. For the RYGB, the pouch was 25–30 mL in size, and the Roux limb lengths ranged between 100 and 125 cm. Although the common channel measurement was not documented in all cases, the goal was to maintain a total alimentary limb length (Roux and common channel) of at least 400 cm.

### Data collection

Clinical baseline information, including the precursor to ostomy, smoking status, preoperative diabetes mellitus type II, systemic arterial hypertension, dyslipidemia, gastroesophageal reflux disease (GERD), history of myocardial infarction, atrial fibrillation, previous pulmonary embolism, chronic obstructive pulmonary disease, immunosuppression status, and sleep apnea.

Intraoperative notes were screened for intraoperative complications. For short-term complications, all records were screened up to 30 days after surgery, including emergency department visits and infusion center visits. Long-term complications included reoperation, anastomotic stricture, hernia, marginal ulceration, gastrointestinal leakage, dumping syndrome, short bowel syndrome, and small intestinal bacterial overgrowth. In addition, the nutritional profile and ostomy output were also observed.

## Results

### Baseline characteristics

Matching the ICD and CPT codes resulted in 59 patients. However, after a thorough chart review, only six patients were found to have an ileostomy or colostomy constructed before bariatric surgery. All cases were operated laparoscopically. Demographics and obesity-related comorbidities are summarized in Table 1. Four patients (66%) were women, the mean age was 58 (± 11.86) years, and the mean preoperative body mass index was 41.58 (± 16.34) kg/m^2^. SG was performed in four (66%) patients and RYGB in two (33%).

**Table 1.** Demographics and Clinical Baseline

Factor	Overall
*N*	6
Age, mean (SD)	58 (± 11.86)
BMI, mean (SD)	41.59 (± 16.34)
Gender: female, *n* (%)	4 (66.67)
Race, *n* (%)	
White	5 (83.34)
Other	1 (16.67)
Diabetes mellitus type II	4 (66.67)
Hyperlipidemia	4 (66.67)
Hypertensive requiring medication	4 (66.67)
Current smoker within 1 year	0 (0)
Obstructive sleep apnea	3 (50.00)
Therapeutic anticoagulation	2 (33.34)
Immunosuppression	2 (33.34)
GERD requiring medication	5 (83.34)
ASA class, *n* (%)	
3	5 (83.34)
4	1 (16.67)
Year of operation, *n* (%)	
2018	3 (50.00)
2020	1 (16.67)
2021	2 (33.34)
Surgical procedure, *n* (%)	
Roux-en-Y gastric bypass	2 (33.34)
Sleeve gastrectomy	4 (66.67)

ASA, American Society of Anesthesiologists; BMI, body mass index; GERD, gastroesophageal reflux disease; SD, standard deviation.

### Ostomy

Three patients had a colostomy, and three had an ileostomy, as summarized in Table 2. Mean time from ostomy creation to surgery was 11.67 (± 8.98) years. All patients had a closed/ended ostomy. From two patients who underwent RYGB, one had an ileostomy and one a colostomy. Of the SG group, two patients had an ileostomy, and the other two had a colostomy. From four patients with a parastomal hernia, only one had to be addressed intraoperatively at the time of bariatric surgery (primary repair). For the cause of ostomy in the SG, two were due to trauma, one due to Crohn's disease, and the other one due to ulcerative colitis. In the RYGB group, one ostomy was due to colon cancer, and the other one was due to scleroderma leading to rectal prolapse with severe fecal incontinence.

**Table 2.** Ostomy

Factor	Overall
*N*	6
Ileostomy, *n* (%)	3 (50.00)
Colostomy, *n* (%)	3 (50.00)
Years of ostomy before surgery, mean (SD)	11.67 (±8.98)
Ended ostomy, *n* (%)	6 (100)
Parastomal hernia, *n* (%)	4 (66.67)
Cause of ostomy, *n* (%)	
Trauma	2 (33.34)
IBD	2 (33.34)
Cancer	1 (16.67)
Scleroderma/fecal incontinence	1 (16.67)

IBD, inflammatory bowel disease.

### Postoperative follow-up and complications

Table 3 summarizes the postoperative follow-up and complications. The mean follow-up was 23 (± 16.43) months. The mean percentage of total weight loss (%TWL) on the follow-up period was 15.50% (± 11.77).

**Table 3.** Postoperative Follow-Up

Factor	Overall
*N*	4
Mean follow-up (SD)	23 months (16.43)
Mean percentage of weight loss (SD)	15.50% (11.77)
Upto 30 days complications, *n* (%)	
Postoperative ED visit	1 (25.00)
Conversion to open	0 (00)
Visits to an infusion center	0 (00)
Upto 3 years complications, *n* (%)	
Bariatric revision—SG to RYGB	1 (25.00)
Dumping	1 (25.00)
Anastomotic stricture	1 (25.00)

ED, emergency department; RYGB, Roux-en-Y gastric bypass; SG, sleeve gastrectomy.

Up to 30 days of follow-up, there were no readmissions due to dehydration or outpatient fluid infusions, and only one patient had an emergency department visit for acrochordon removal. Long-term complications included one case of intractable reflux leading to conversion from SG to RYGB. Moreover, one RYGB patient presented with anastomotic stricture and dumping. The stricture was treated with one endoscopic dilation after the surgery and the dumping with dietary counseling. None of the patients had increased postoperative ostomy output, dehydration, or needed fluid infusion. No malnutrition (assessed through routine postoperative bariatrician clinic notes) was noted during the follow-up period.

## Discussion

In this report, no short-term complications were encountered in patients with a history of stoma undergoing bariatric surgery. In the long term, one patient had dumping syndrome and an anastomotic stricture managed with dilation, and one patient underwent revisional surgery. There were no conversions to open surgery or episodes of dehydration requiring medical care. Interestingly, this particular population behaved similarly to patients without a stoma.^11^

We had expected these patients to have a higher stoma output relative to their baseline, especially in the early postoperative period; however, we found no changes in the stomal output in the short-term evaluation.^12^ A possible explanation could include the development of adaptation mechanisms because these patients had long-standing ostomies.^13^ In fact, patients develop hypertrophy and hyperplasia of the remaining intestinal length early after the procedure.^14^ Over time, slower intestinal transit leads to better absorption of nutrients,^15^ and small bowel electrolyte transport changes lead to a proportional response to fluids.^16^

Only a single case report with a patient undergoing RYGB with a pre-existing ostomy had an uneventful 12-month postoperative course.^10^ Similarly, our data showed a relatively low rate of adverse events mainly related to the procedure as only one patient eventually required surgical revision. Despite both procedures being safe and effective,^4,5^ careful decision-making to avoid multiple surgeries is more relevant to this population. In our report, a patient with mild self-reported GERD symptoms underwent SG. One of the factors affecting the decision-making process was the lack of data about the feasibility of RYGB in this particular population. This patient ended up requiring conversion to RYGB due to the severity of the reflux, and had an uneventful postoperative course, and witnessed amelioration in his reflux symptoms.^17–20^

Obesity is related to an increase in the incidence of parastomal hernias.^21,22^ In addition, recurrence rates are higher after hernia repair in patients with obesity.^23,24^ In our report, four patients had parastomal hernias. Our approach to parastomal hernias is similar to our approach to any ventral hernia at the time of bariatric surgery. We attempt to leave the hernia content within the hernia sac if possible and deal with the hernia repair later once weight loss is achieved. However, if hernia contents were reduced, primary closure of the defect should be performed to prevent bowel incarceration postoperatively, thus leading to obstruction and potentially proximal leak.

This report has several limitations. Initially, the small sample size greatly reduces the chances of generalizability of our results. In addition, the lack of a control group introduces several biases. Finally, the relatively short follow-up with a mean follow-up of 23 months is a limitation. The fundamentally different underlying diagnosis that leads to ostomy also introduces biases. Further investigations with bigger numbers, stratified analysis, and a longer follow-up are required to better understand the postoperative behaviors pertaining to this specific population.

## Conclusion

Bariatric surgery in the setting of pre-existing ostomy is technically feasible with a reasonable short-term safety profile. Further investigations with bigger numbers and a longer follow-up are required to better understand the postoperative behaviors pertaining to this specific population.
